# Early Diverging Insect-Pathogenic Fungi of the Order Entomophthorales Possess Diverse and Unique Subtilisin-Like Serine Proteases

**DOI:** 10.1534/g3.118.200656

**Published:** 2018-08-21

**Authors:** Jonathan A. Arnesen, Joanna Małagocka, Andrii Gryganskyi, Igor V. Grigoriev, Kerstin Voigt, Jason E. Stajich, Henrik H. De Fine Licht

**Affiliations:** * Section for Organismal Biology, Department of Plant and Environmental Sciences, University of Copenhagen, Denmark; † Department of Biology, Duke University, Durham, North Carolina, USA; ‡ US Department of Energy Joint Genome Institute, Walnut Creek, California, USA; § Jena Microbial Resource Collection (JMRC), Leibniz Institute for Natural Product Research and Infection Biology - Hans Knoell Institute, Adolf-Reichwein-Str.23, 07745 Jena, Germany; ** Institute of Microbiology, Friedrich Schiller University, Neugasse 25, 07743 Jena, Germany; †† Department of Plant Pathology and Microbiology, University of California, Riverside, California

**Keywords:** Subtilase, insect-pathogen, early-diverging fungi, proteases, phylogenomics

## Abstract

Insect-pathogenic fungi use subtilisin-like serine proteases (SLSPs) to degrade chitin-associated proteins in the insect procuticle. Most insect-pathogenic fungi in the order Hypocreales (Ascomycota) are generalist species with a broad host-range, and most species possess a high number of SLSPs. The other major clade of insect-pathogenic fungi is part of the subphylum Entomophthoromycotina (Zoopagomycota, formerly Zygomycota) which consists of high host-specificity insect-pathogenic fungi that naturally only infect a single or very few host species. The extent to which insect-pathogenic fungi in the order Entomophthorales rely on SLSPs is unknown. Here we take advantage of recently available transcriptomic and genomic datasets from four genera within Entomophthoromycotina: the saprobic or opportunistic pathogens *Basidiobolus meristosporus*, *Conidiobolus coronatus*, *C. thromboides*, *C. incongruus*, and the host-specific insect pathogens *Entomophthora muscae* and *Pandora formicae*, specific pathogens of house flies (*Muscae domestica*) and wood ants (*Formica polyctena*), respectively. In total 154 SLSP from six fungi in the subphylum Entomophthoromycotina were identified: *E. muscae* (n = 22), *P. formicae* (n = 6), *B. meristosporus* (n = 60), *C. thromboides* (n = 18), *C. coronatus* (n = 36), and *C. incongruus* (n = 12). A unique group of 11 SLSPs was discovered in the genomes of the obligate biotrophic fungi *E. muscae*, *P. formicae* and the saprobic human pathogen *C. incongruus* that loosely resembles bacillopeptidase F-like SLSPs. Phylogenetics and protein domain analysis show this class represents a unique group of SLSPs so far only observed among Bacteria, Oomycetes and early diverging fungi such as Cryptomycota, Microsporidia, and Entomophthoromycotina. This group of SLSPs is missing in the sister fungal lineages of Kickxellomycotina and the fungal phyla Mucoromyocta, Ascomycota and Basidiomycota fungi suggesting interesting gene loss patterns.

Insect pathogenic fungi use a broad array of enzymes to penetrate the host cuticle and gain entry to the soft tissues inside (Charnley 2003; St. Leger *et al.*, 1986b). In many cases, serine proteases are among the first enzymes to be secreted in the early stages of infection in order to cleave and open up chitin-associated proteins in the procuticle (St. Leger *et al.*, 1986a; Vega *et al.*, 2012), which later is followed by extensive lipase and chitinase enzymatic secretions (Charnley 2003). In particular, subtilisin-like serine proteases (SLSPs) have been considered important virulence factors in pathogenic fungi (Muszewska *et al.*, 2011). The first SLSPs from insect pathogenic fungi were identified in *Metarhizium anisopliae* (ARSEF2575), which secretes SLSPs as some of the key proteases during fungal growth on insect cuticle (Charnley 2003; St. Leger *et al.*, 1986a). Comparative genomic approaches have identified significant expansions of the SLSP gene family in the genus *Metarhizium* (Bagga *et al.*, 2004; Hu *et al.*, 2014), the insect pathogenic fungus *Beauveria bassiana* (Xiao *et al.*, 2012), two nematode-trapping fungi *Monacrosporium haptotylum* and *Arthrobotrys oligospora* that are able to penetrate the chitinaceous cell wall of soil nematodes (Meerupvati *et al.* 2013), and dermatophytic fungi such as *Arthroderma benhamiae* and *Trichophyton verrucosum* that can cause nail and skin infections in humans and animals (Burmester *et al.*, 2011; Desjardins *et al.*, 2011; Martinez *et al.*, 2012; Sharpton *et al.*, 2009). Fungi that are able to utilize chitin-rich substrates, including many insect pathogenic fungi, thus appear to often be associated with a diversified and expanded set of SLSPs.

Although SLSPs are expanded among insect pathogenic fungi, this group of proteases is ubiquitous among eukaryotic organisms. Most SLSPs are secreted externally or localized to vacuoles, and especially in saprobic and symbiotic fungi SLSPs constitute an important component of the secretome (Li *et al.*, 2017). According to the MEROPS peptidase classification, the S8 family of SLSPs together with the S53 family of serine-carboxyl proteases make up the SB clan of subtilases (Rawlings *et al.*, 2016). The S8 family of SLSPs is characterized by an Asp-His-Ser catalytic triad (DHS triad), which forms the active site and is further divided into two subfamilies S8A and S8B. Subfamily S8A contains most S8 representatives, including the well-known Proteinase K enzyme that is widely used in laboratories as a broad-spectrum protease. The S8B SLSPs are kexins and furins which cleave peptides and protohormones (Jalving *et al.*, 2000; Muszewska *et al.*, 2017, 2011). Based on characteristic protein domain architectures and protein motifs surrounding the active site residues, the large S8A subfamily of SLSPs is further divided into several groups such as proteinase-K and pyrolysin. Besides these two major groups of proteinase K-like and pyrolisin subfamilies, six new groups of subtilase genes designated *new 1* to *new 6* have recently been found (Li *et al.*, 2017; Muszewska *et al.*, 2011). The analysis of fungal genome data from a wide taxonomic range has shown that the size of the proteinase K gene family has expanded independently in fungi pathogenic to invertebrates (Hypocreales) and vertebrates (Onygenales) (Muszewska *et al.*, 2017; Sharpton *et al.*, 2009). Closely related invasive human-pathogenic fungi, however, do not show the same expansions and related pathogens and non-pathogens can show the same expansions (Muszewska *et al.*, 2011; Whiston and Taylor 2016). This suggests that the number of SLSPs that a fungus possess is not directly related to pathogenicity, but instead is associated with the use of dead or alive animal tissue as growth substrate (Li *et al.*, 2017; Muszewska *et al.*, 2011).

Most anamorphic insect-pathogenic fungi in the order Hypocreales (Ascomycota) are generalist species with a broad host-range capable of infecting most major orders of insects (*e.g.*, *M. robertsii* and *B. bassiana*) or specific to larger phylogenetic groups (*e.g.*, the locust-specific *M. acridum* or the coleopteran pathogen *B. brongniartii*) (Boomsma *et al.*, 2014; Hu *et al.*, 2014). The above-mentioned inferences of fungal SLSP evolution rely almost exclusively on insights from Ascomycota, and consequently have strong sampling bias toward generalist insect-pathogenic fungi. In contrast, the other major clade of insect-pathogenic fungi in the subphylum Entomophthoromycotina (Zoopagomycota, formerly Zygomycota) consists almost exclusively of insect-pathogens and many are extremely host-specific, naturally only infecting a single or very few host species (Spatafora *et al.*, 2016). The dearth of genomic data for Entomophthoromycotina has previously precluded their inclusion in comparative genomic analyses (De Fine Licht *et al.*, 2016; Gryganskyi and Muszewska 2014). Here we take advantage of recently available transcriptomic and genomic datasets from four genera within Entomophthoromycotina: the saprobic *Basidiobolus meristosporus*, the saprobic and opportunistic pathogens, *Conidiobolus coronatus*, *C. thromboides*, *C. incongruus*, and the host-specific insect pathogens *Entomophthora muscae* and *Pandora formicae*, specific pathogens of house flies (*Muscae domestica*) and wood ants (*Formica polyctena*), respectively. We use phylogenetics and protein domain analysis to show that the obligate biotrophic fungi *E. muscae*, *P. formicae* and the saprobic human pathogen *C. incongruous*, in addition to more “classical” fungal SLSPs, harbor a unique group of SLSPs that loosely resembles bacillopeptidase F-like SLSPs.

## Materials and Methods

### Sequence database searches for subtilisin-Like serine proteases

We identified putative subtilisin-like serine proteases (SLSPs) from six fungi in the subphylum Entomophthoromycotina: *Entomophthora muscae*, *Pandora formicae*, *Basidiobolus meristosporus*, *Conidiobolus coronatus*, *C. incongruus* and *C. thromboides*. First, protein family (pfam) domains were identified in the *de-novo* assembled transcriptomes of *E. muscae* KVL-14-117 (De Fine Licht *et al.*, 2017) and *P. formicae* (Małagocka *et al.*, 2015) using profile Hidden Markov Models with hmmscan searches (E-value < 1e-10) against the PFAM-A database ver. 31.0 using HMMER ver. 3 (Eddy 1998; Finn *et al.*, 2016). All sequences in the transcriptome datasets containing the S8 subtilisin-like protease domain (PF00082) were identified and included in further analyses. Second, all sequences that contain the PF00082 domain were obtained from the genomes of *B. meristosporus* CBS 931.73 (Mondo *et al.*, 2017), *C. coronatus* NRRL28638 (Chang *et al.*, 2015), and *C. thromboides* FSU 785 from the US Department of Energy Joint Genome Institute *MycoCosm* genome portal (http://jgi.doe.gov/-fungi). Third, predicted coding regions in the genome sequence of *C. incongruus* CDC-B7586 (Chibucos *et al.*, 2016), were searched for the presence of the S8 subtilisin-like protease domain (PF00082) as described above.

Sequences encoding an incomplete Asp-His-Ser catalytic triad (DHS triad) characteristic of S8 family proteases were regarded as potential pseudogenes and excluded from further analysis. Although a subset may represent neofunctionalizations, the risk of including false-positive SLSPs in down-stream analyses were considered too high. A preliminary protein alignment made with ClustalW (Larkin *et al.*, 2007) using default parameters and construction of a Neighbor-Joining tree using Geneious 4.8.5 (Kearse *et al.*, 2012) revealed a highly divergent group of *P. formicae* SLSP-sequences that had significant homology with insect proteases (blastp, E-value < 1e-6, ncbi-nr protein database, accessed June 2017). These putative insect-sequences likely originate from the ant host, *Formica polyctena*, and represents host contamination that were not filtered out from the dual-RNAseq reads used to construct the *P. formicae* transcriptome (Małagocka *et al.*, 2015). These divergent sequences were therefore removed and excluded from further analysis.

### Protein domain architecture and sequence clustering

The domain architectures of all putative SLSPs identified within Entomophthoromycotina were predicted using Pfam domain annotation. The presence of putative signal peptides for extracellular secretion were predicted using SignalP (Petersen *et al.*, 2011). A Markov Clustering Algorithm (MCL) was used to identify clusters of similar proteins among putative SLSPs identified within Entomophthoromycotina. Clustering using MCL is based on a graph constructed by an all-vs-all-BLAST of SLSPs (BLASTP, E-value < 1e^-10^). The Tribe-MCL protocol (Enright *et al.*, 2002) as implemented in the Spectral Clustering of Protein Sequences (SCPS) program (Nepusz *et al.*, 2010) was used with *inflation* = 2.0. The *inflation* parameter is typically set between 1.2 – 5.0 (Nepusz *et al.*, 2010), and controls the “tightness” of the sequence matrix, with lower values leading to fewer clusters and higher values to more sequence clusters. To putatively assign protease function to the newly identified Entomophthoromycotina sequence clusters, the Tribe-MCL protocol was used to identify clusters of similar proteins between the putative SLSPs identified within Entomophthoromycotina and 20,806 protease sequences belonging to the peptidase subfamily S8A obtained from the MEROPS database, accessed November 2017 (Rawlings *et al.*, 2016). Investigation of the MEROPS protease sequences that clustered together with the identified Entomophthoromycotina sequence clusters allowed putative protease holotype information to be assigned to the identified clusters.

### Phylogenetic analysis

All identified putative Entomophthoromycotina SLSP coding nucleotide sequences were aligned in frame to preserve codon structure using MAFFT (Larkin *et al.*, 2007). Unreliable codon-columns with a Guidance2 score below 0.90 in the multiple sequence alignment were removed (Penn *et al.*, 2010). The best model for phylogenetic analysis was selected by running PhyML with *GTR* as substitution model and with-or-without Gamma parameter and a proportion of invariable sites (Guindon and Gascuel 2003). The optimal substitution model based on the Bayesian Information Criterion (BIC) score (*GTR+G*) was determined using TOPALI 2.5 (Milne *et al.*, 2009) and used in maximum likelihood analysis using RaxML with 10,000 bootstrap runs (Stamatakis 2014).

To identify branches that potentially contain signatures of positive selection among SLSP sequences we used maximum likelihood estimates of the dN/dS ratio (ω) for each site (codon) along protein sequences. A specific lineage (branch) was tested independently for positive selection (ω > 1) on individual sites by applying a neutral model that allows ω to vary between 0 – 1 and a selection model that also incorporates sites with ω > 1 using the software *codeml* implemented in PAML 4.4 (Yang 2007). Statistical significance was determined with a likelihood ratio test of these two models for the tested lineage.

To infer phylogenetic relationship of identified putative Entomophthoromycotina SLSPs with fungal SLSPs from other invertebrate-associated ascomycete fungi, two approaches were used. First, all S8A SLSPs in the MEROPS database from the insect-pathogenic genera *Metarhizium* (n = 240), *Cordyceps* (including *Beauveria*) (n = 44), *Ophiocordyceps* (n = 11), and the nematode-trapping genera *Arthrobotrys* (n = 33), and *Monachrosporium* (n = 8), were clustered by sequence identity with the identified SLSPs from Entomophthoromycotina (n = 152) using the MCL approach previously described. Second, from the three identified SLSP groups (A, B, and C) within Entomophthoromycotina, 10 representative group A SLSPs and all sequences from group B (n = 11) and group C (n = 11) were searched against the entire S8A MEROPS database (blastp, e-value = 1e-10). The non-redundant top-ten hits from this search (n = 149) were aligned with the Entomophthoromycotina query SLSPs (n = 32) and representative invertebrate-associated SLSPs from the clustering analysis (n = 350) using MAFFT. Unreliable columns with a Guidance2 score below 0.80 in the multiple sequence alignment were removed (Penn *et al.*, 2010). The optimal protein substitution model based on the Bayesian Information Criterion (BIC) score (*LG+i*) was determined using ModelFinder (Kalyaanamoorthy *et al.*, 2017) and used in maximum likelihood analysis using RaxML with 100 bootstrap runs (Stamatakis 2014).

### Data availability

All data used are available through the US Department of Energy Joint Genome Institute *MycoCosm* genome portal (http://jgi.doe.gov/-fungi), or has been previously published (Małagocka *et al.*, 2015; Chibucos *et al.*, 2016; De Fine Licht *et al.*, 2017). SLSP sequence data and Tribe-MCL outputs analyzed in this manuscript are provided as a zip-compressed single file ArnesenEtalSupplementaryData.zip in the supplementary material. Supplemental material available at Figshare: https://doi.org/10.25387/g3.6949037.

## Results

**Figure 1:**
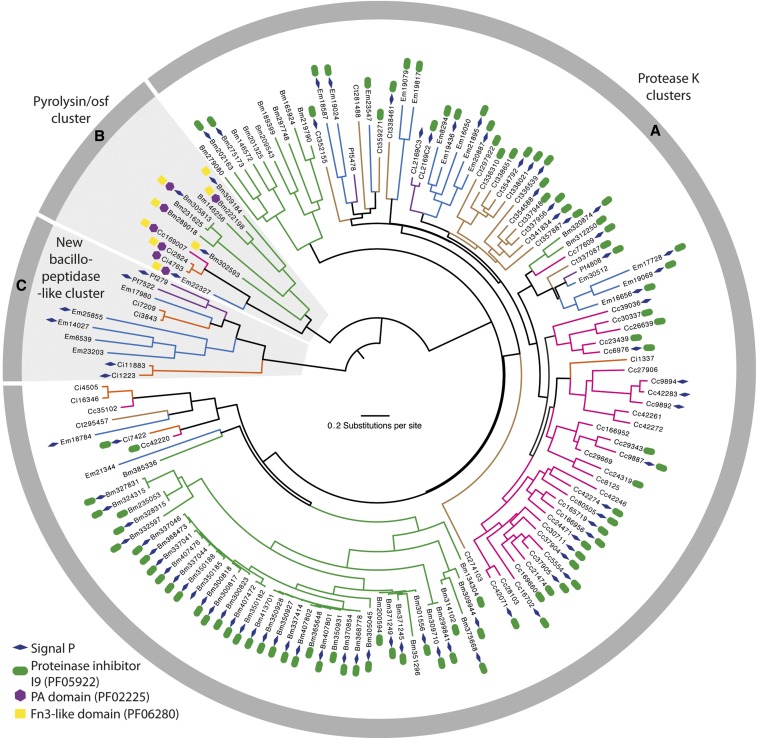
Maximum likelihood phylogeny calculated with RAxML and based on a 2,379 bp alignment of 152 subtilisin-like serine protease codon nucleotide sequences from Entomophthoromycotina that contain the peptidase S8/S53-subtilisin (PF00082) domain. Branches are colored for eachs species as *Entomophthora muscae* (Blue), *Pandora formicae* (Purple), *Conidiobolus coronatus* (Pink), *C. thromboides* (brown), *C. incongruus* (orange), and *B. meristoporus* (Green). For each SLSP, the accession number and protein domains additional to PF00082 are shown. The three identified clusters: Protease K cluster (A), Pyrolysin/osf protease cluster (B), and the new bacillopeptidase-like Entomophthorales cluster (C), are marked in the gray circle surrounding the tree and with gray background for cluster B and C.

We identified 154 SLSP sequences from six fungi in the subphylum Entomophthoromycotina: *E. muscae* (n = 22), *P. formicae* (n = 6), *B. meristosporus* (n = 60), *C. thromboides* (n = 18), *C. coronatus* (n = 36), and *C. incongruus* (n = 12). Close inspection of the active site residues revealed two *C. incongruus* sequences (Ci7229 and Ci12055), which contained the active site DHS residues in the motifs Asp-Asp-Gly, His-Gly-Thr-Arg, and Gly-Thr-Ser-Ala/Val-Ala/Ser-Pro characteristic of the S8B subfamily of S8 proteases. These two sequences also contained the P domain (PF01483) indicating that they are S8B kexin proteases. All other 152 identified Entomophthoromycotina S8 protease sequences contained active site residues closely resembling the motifs Asp-Thr/Ser-Gly, His-Gly-Thr-His, and Gly-Thr-Ser-Met-Ala-Xaa-Pro characteristic of the S8A subfamily. Cluster analysis of these 152 S8A-protease sequences identified three groups of proteins that were designated as group A, B and C, with 130, 11 and 11 sequences in each cluster, respectively (Figure 1). These group memberships do not change when the Tribe-MCL *inflation* parameters varied within a range of 1.5 – 6.0 suggesting that these three groups were clearly distinct. Phylogenetic analysis of the identified 152 S8A SLSPs using maximum likelihood methods also recovered the same three distinct lineages (Figure 1). Evidence of positive selection acting on specific enzyme residues on the branches leading to these clusters were not detected with branch-site tests (2ΔlnL > 1.53, *P* > 0.217).

**Figure 2:**
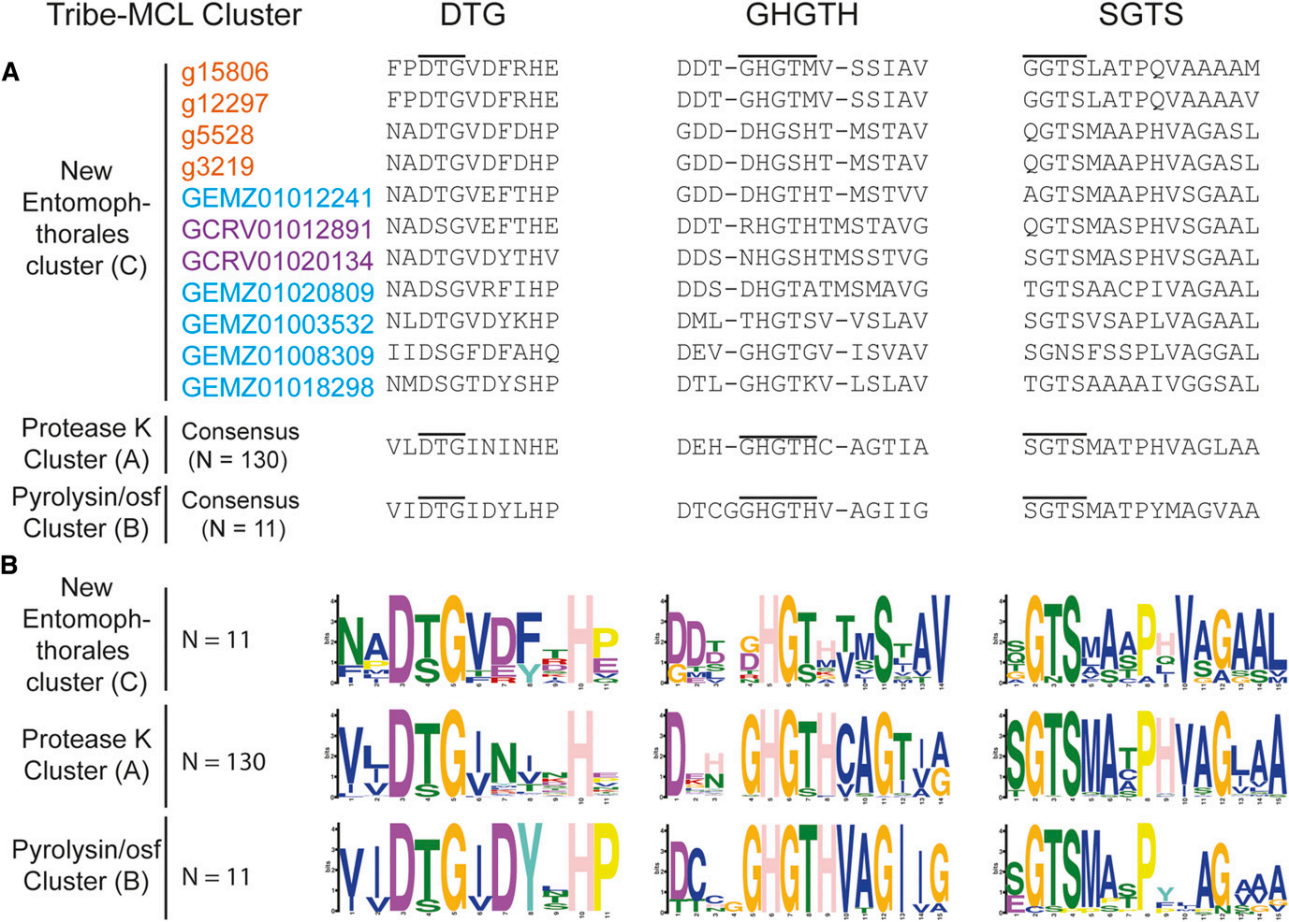
Active site and domain co-occurrence variability of the three Tribe-MCL clusters identified among 152 Entomophthoromycotina subtilisin-like serine proteases. The columns DTG, GHGTH, and SGTS represents the closest amino acid sequence for each of the amino acids from the DHS catalytic triad. A. Amino acid alignment of the active site residues for the three identified groups (A-C) of SLSPs within Entomophthoromycotina. Accession codes are color coded as: Orange – *C. incongruus*, Blue – *E. muscae*, and Purple – *P. formicae*. B. Sequence motifs of the active site residues for each group.

To further characterize the three SLSP groups, the protein domain architecture of each of the 152 protease sequences were analyzed. The presence of a proteinase-associated (PA, pfam:PF02225) domain was only found in Group B that strongly suggested this cluster with 11 members is comprised of pyrolisin and osf proteases (Muszewska *et al.*, 2011). An additional Tribe-MCL cluster analysis (inflation = 1.2) of the 152 Entomophthoromycotina and all S8A proteases in the MEROPS database clustered these 11 Entomophthoromycotina sequences into a group of 779 MEROPS proteases. This group of proteases contained 42 members of the fungal S08.139 (PoSl-(*Pleurotus ostreatus*)-type peptidase) holotype (Supplementary data). The protein domain architecture of group A contained 130 Entomopthoromycotina SLSPs, and many of these 130 SLSPs contained a secretory signal and/or a peptidase inhibitor (Inhibitor_I9, pfam: PF05922) domain. The 130 Entomophthoromycotina SLSPs clustered with 3,046 MEROPS proteins of well-known fungal entomopathogenic protease holotypes (Supplementary data), including cuticle-degrading peptidase of nematode-trapping fungi (S08.120), cuticle-degrading peptidase of insect-pathogenic fungi in the genus *Metarhizium* (S08.056), and subtilisin-like peptidase 3 (*Microsporum*-type; S08.115). The 130 Entomophthoromycotina Group A SLSPs thus belong to the common Proteinase K group of S8A proteases (Muszewska *et al.*, 2011) based on conservation of the active site residues (Figure 2), and cluster membership of the MEROPS Tribe-MCL analysis.

**Figure 3:**
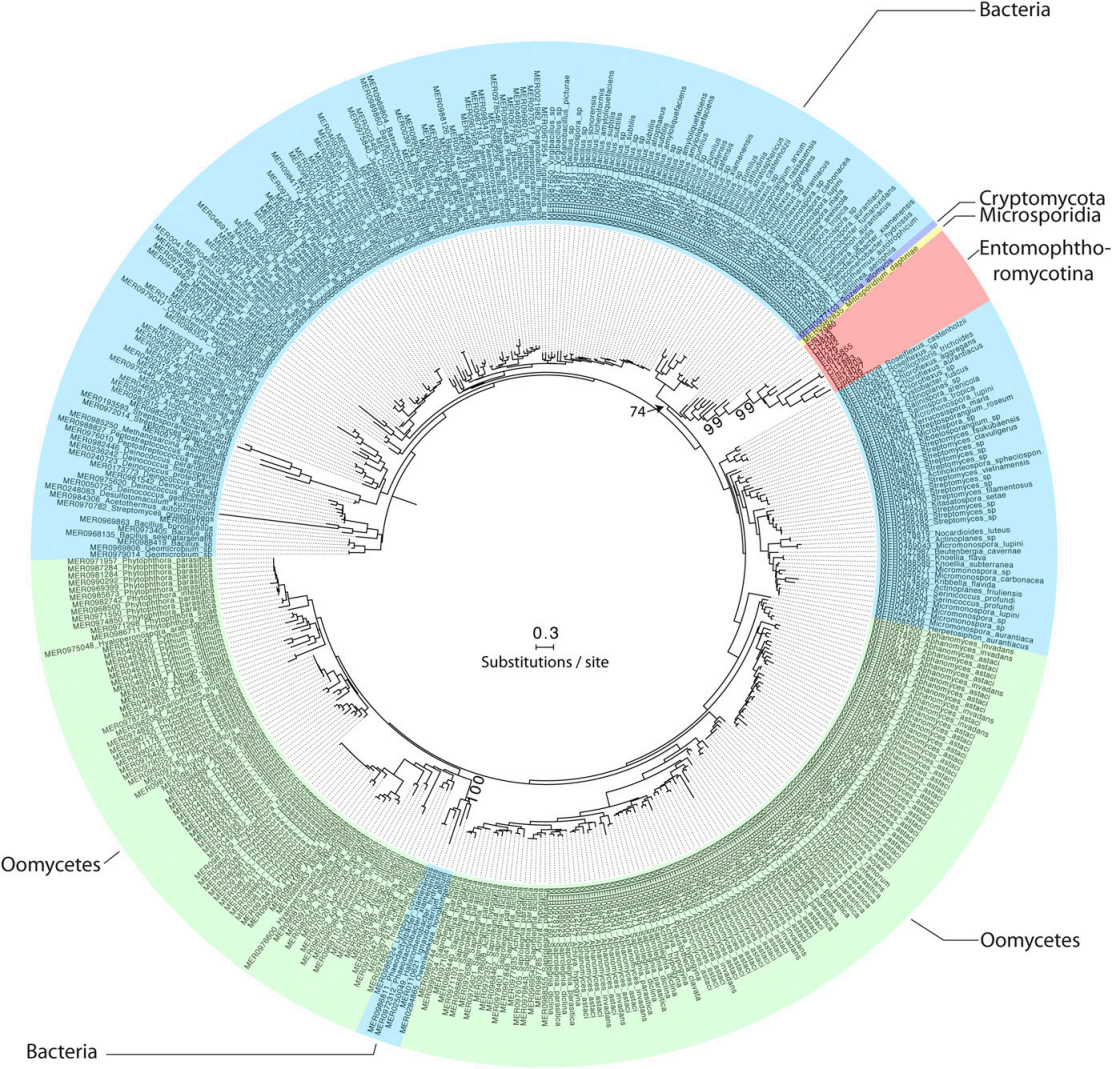
Mid-point rooted maximum likelihood phylogeny calculated with RAxML and based on a (479 amino acid) alignment of 413 protein subtilisin-like serine protease sequences, which belonged to group C in the Tribe-MCL analysis (see text for details). Bootstrap values >70 from 1000 iterations are shown for non-terminal deeper nodes.

**Figure 4:**
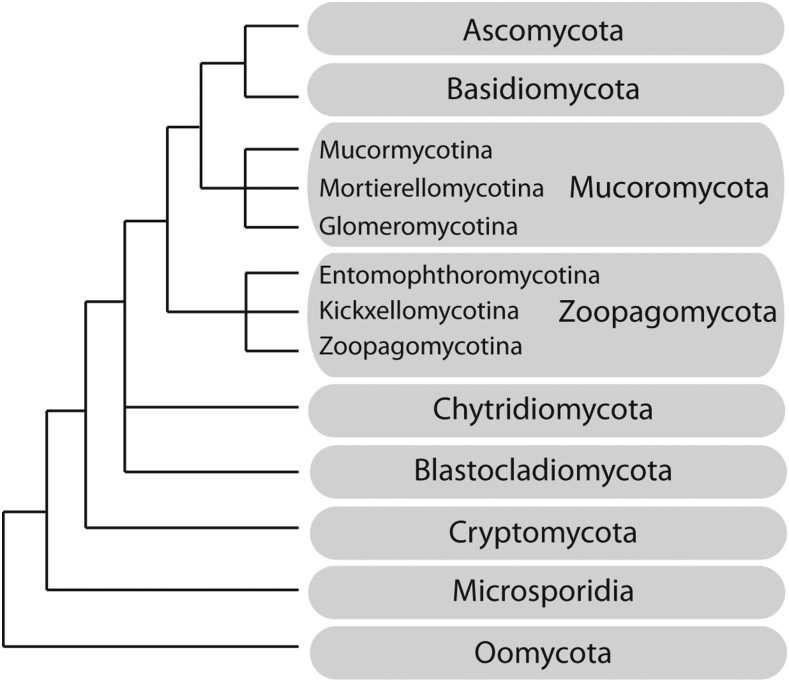
Schematic phylogeny and classification of the early-diverging fungi and related taxonomic groups principally based on Spatafora *et al.* (2016). Branch lengths are not proportional to genetic distances.

**Figure 5:**
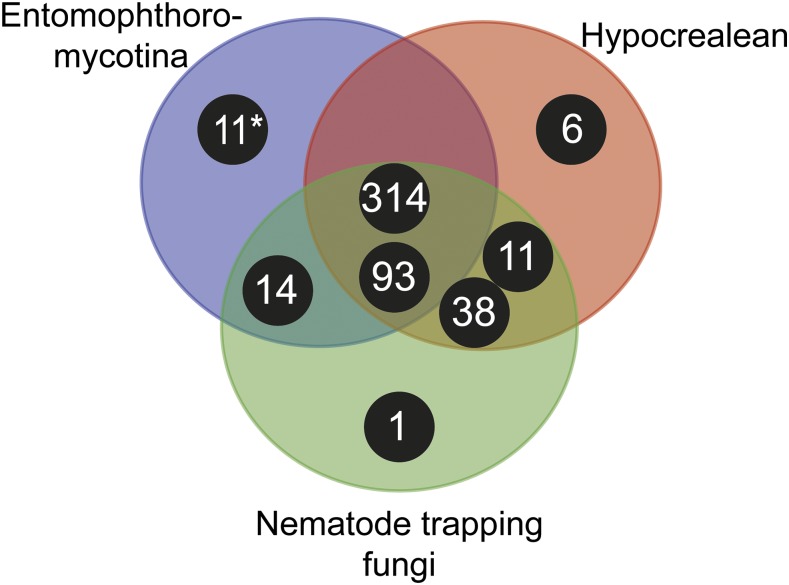
Venn diagram showing taxonomic distribution of subtilisin-like serine protease clusters of major insect and nematode-pathogenic fungal genera. Black circles correspond to clusters with number of S8A proteases for each cluster, and the placement within the Venn diagram correspond to the taxonomic groups contributing sequences to a specific cluster. The asterisk (*) marks the 11 members of the new bacillo-peptidase like cluster C described within the order Entomophthorales (subphylum: Entomophthoromycotina). Entomophthoromycotina encompasses SLSP’s found in the genera: *Basidiobolus*, *Conidiobolus*, *Entomophthora*, and *Pandora*. Hypocrealean entities consist of SLSP’s in the MEROPS database from the genera: *Cordyceps*, *Metarhizium*, *Ophiocordyceps*, and Nematode trapping fungi are MEROPS SLSP’s found in the genera: *Arthrobotrys* and *Monacrosporium*.

**Figure 6:**
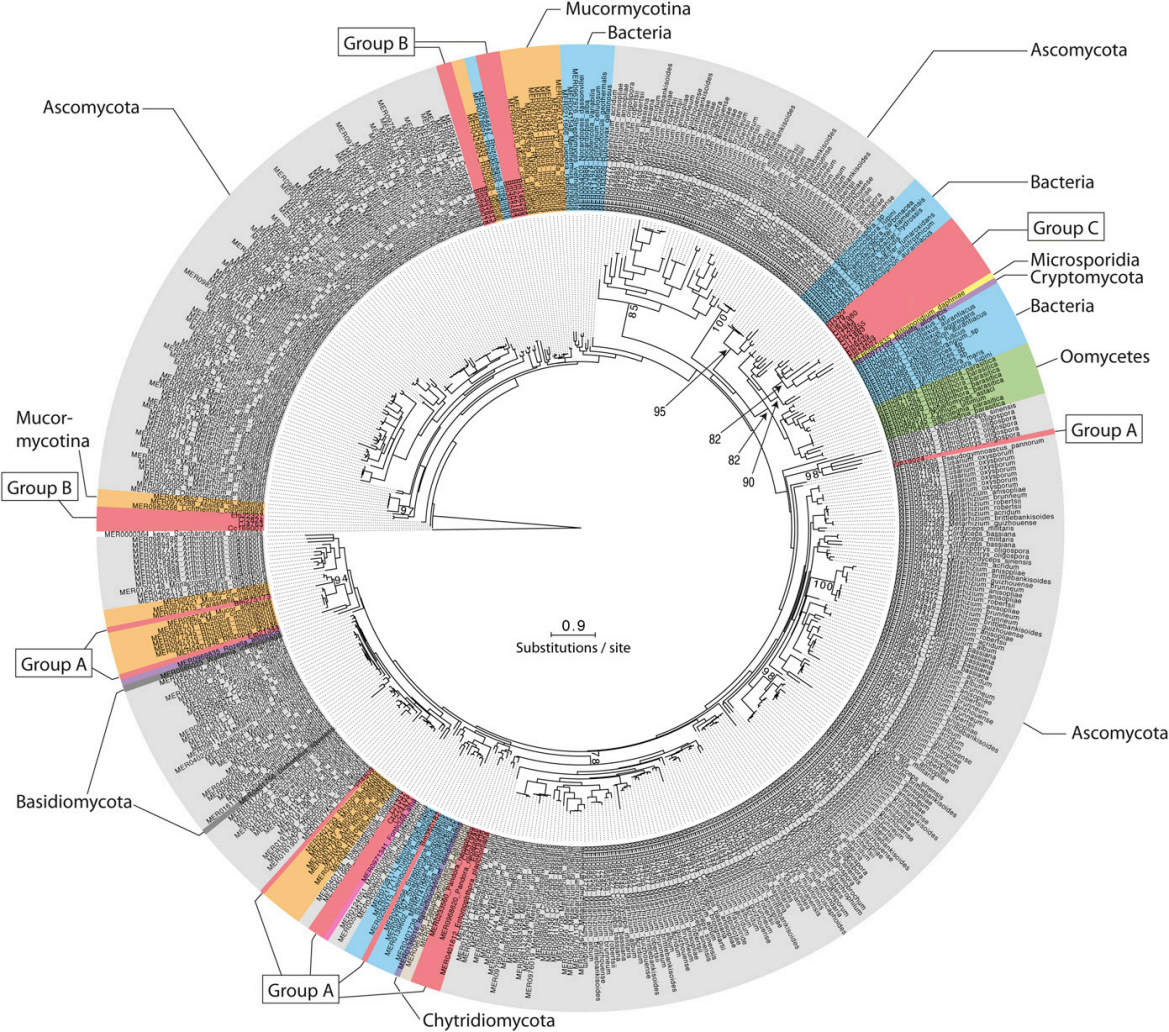
Maximum likelihood phylogeny calculated with RAxML and based on a (276 amino acid) alignment of 500 protein subtilisin-like serine protease sequences. SLPSs within Entomophthoromyctina from group B and C, and 10 representative SLSPs from group A are included together with the 10 most similar SLSPs in the MEROPS database for each group A, B, and C, respectively. To show the relationship between the three Entomophthoromycotina SLSP groups, representative SLSPs from insect-pathogenic and nematode-trapping fungi were included (see text for details). Bootstrap values >70 from 100 iterations are shown for non-terminal nodes. The tree is rooted with the S8B subfamily type Kexin from *S. cerevisiae* (UniProt: P13134)

The MEROPS Tribe-MCL analysis identified a third group (C) of 11 Entomophthoromycotina SLSPs, which only contained members from the order Entomophthorales and clustered with 402 MEROPS proteins (Supplementary data). Of these, 386 were classified as un-assigned subfamily S8A peptidases (S08.UPA) and the remaining 15 assigned to the bacillopeptidase F holotype (S08.017; Supplementary data). All 402 MEROPS proteases in this group originated from either Bacteria or Oomycetes, except for two proteases from the Fungi *Rozella allomycis* (Cryptomycota) and *Mitosporidium daphnia* (Microsporidia), respectively (Figure 3, 4, Supplementary data). The group C Entomophthorales SLSPs clustered as sister to these two Cryptomycota and Microsporidia proteins with strong support (ML bootstrap value = 99) in the phylogenetic analysis of the protein sequences (Figure 3), in concordance with this group of SLSPs being an outlier from all other previously known fungal S8A SLSPs. The monophyly of group C Entomophthorales SLSPs together with Cryptomycota and Microsporidia proteins were further supported in the phylogenetic analysis including all three Entomophthoromycotina groups (Figure 6) and when compared to invertebrate-associated ascomycete SLSPs (Figure 5).

## Discussion

Subtilisin-like serine proteases (SLSPs) have many roles in fungal biology and are known to be involved in host–pathogen interactions. Independent expansion of copy number and diversification of SLSPs is widespread among animal pathogenic Dikarya (Ascomycota and Basidiomycota) (Li *et al.*, 2017). The repeated expansion of SLSPs among the generalist insect-pathogenic hypocrealean fungi has been interpreted as an adaptation to enable infection of insect hosts (Muszewska *et al.*, 2011), whereas comparatively little is known about the evolution and diversification of SLSPs among the early diverging fungal clades. To understand the evolution of SLSPs among the vertebrate and arthropod pathogenic fungi in the subphylum Entomophthoromycotina, we searched available genomic and transcriptomic sequence data to identify all Entomophthoromycotina genes with SLSP domains. We found 154 Entomophthoromycotina SLSPs, of which two copies were classified as S8B kexin SLSPs. The remaining 152 S8A SLSPs were clustered by sequence similarity and compared by phylogenetic analysis to show that the majority of the SLSPs (n = 130) are similar to and cluster together with “classical” proteinase-K-like fungal S8A SLSPs (Figure 1). A statistical test for a significant expansion of SLSP copy number among the insect-pathogenic Entomophthoromycotina was not explicitly performed in the present analysis due to uncertainty of total gene numbers from transcriptomic data sets of the specialist insect-pathogens *E. muscae* and *P. formicae*. In the sampled transcriptomes, the number of transcripts is likely larger than the genome gene count due to splice variants, post-transcriptional modifications, and allelic variants assembling into multiple transcripts per gene. In addition, the assembled transcripts only reflect actively transcribed genes expressed in the sampled conditions and time points, and may underestimate the actual number of genes. These confounding factors impact the estimated number of genes and make quantitative comparative analyses of gene family size between transcriptomes unreliable.

We did identify 11 SLSPs that cluster together with 402 un-annotated or Bacillopeptidase F-like SLSPs primarily from bacteria and Oomycetes (Figure 3), but also including two fungal protease sequences from the early-diverging Cryptomycota *R. allomycis* and microsporidium *M. daphnia* lineages (Figure 4). These observations remained consistent even when exploring variation in the inflation parameter, which controls the “tightness” in the cluster analysis. The entomophthoralean and oomycetous S8A SLSPs form separate clades within this 402-sequence cluster of primarily bacterial proteases. Instead, the 11 Entomophthorales SLSPs form a distinct lineage together with the two proteases from *R. allomycis* and *M. daphnia* (Figure 3, 6). This indicates that within this group, the Entomophthorales and Oomycete SLSPs both share a most recent common ancestor with independent bacterial proteases and the 11 entomophthoralean proteases, together with the two SLSPs from Cryptomycota and Microsporidia, are a unique group of proteases exclusive to some of the early diverging fungal lineages.

Functional annotation indicates apparent protease activity based on sequence similarity, but molecular function of the novel 11 SLSPs in group C is unknown. Eight of these SLSPs possess a signal peptide that suggest external secretion and thus indicative of a function on the immediate environment of the fungi, whereas the remaining three SLSPs might not be secreted or represent incomplete sequence models. Apart from the canonical protease S8 domain (PF00082), no other Pfam domains were found among this group C SLSPs. Searches against InterPro databases similarly did not reveal any other protein domains apart from the protease S8 SLSP domain (PRINTS: subtilisin serine protease family (S8) signature (PR00723), InterPro: peptidase S8, subtilisin-related (IPR015500), and ProSitePatterns: serine proteases, subtilase family (PS00138)). Notably, none of these SLSPs contain the proteinase inhibitor i9 domain (PF05922) often found among the classical protease K-like SLSPs (Muszewska *et al.*, 2011). Extensive diversification of the amino acids immediately surrounding the active site residues in the DHS triad (Figure 2), further suggests that the new group C of SLSPs have evolved a different function than the “classical” fungal SLSPs in group A. Out of the six Entomophthoromycotina species analyzed here, only three: *C. incongruus*, *E. muscae* and *P. formicae* within the order Entomophthorales contained members in the new group C SLSPs (Figure 1). The unequal phylogenetic presence of the group C SLSPs could be indicative of specific functions related to niche adaptation. The two insect-pathogenic fungi specialized on house flies (*E. muscae*) and wood ants (*P. formicae*) contain five and two of the novel group C SLSPs, respectively (Figure 1). However, the soil saprobe and opportunistic human pathogen *C. incongruus* also contains four group C SLSPs, which provide evidence that group C SLSPs are unrelated to host-specific evolution of the specialist insect-pathogenic entomophthoralean fungi.

The novel group C SLSPs were absent from the sequenced genome of *C. thromboides*, implying that the uneven taxonomic presence and absence of particular SLSPs within Entomophthoromycotina taxa are unlikely to be due to sequencing or sampling artifacts of data. This is further supported by the absence of group C SLSP S8A-proteases in Basidiomycota or Ascomycota (Figure 3). However, the genomes of ascomycete insect-pathogenic hypocrealean and nematode-trapping fungi within Ascomycota contain different SLSP’s missing in Entomphthoromycotina (Figure 5). The present analysis shows that the two major groups of insect-pathogenic fungi within Ascomycota and Entomophthoromycotina contain a similar complement of SLSPs, but each clade also possesses unique sets of these proteases that most likely evolved independently (Figure 6). Further studies including genomic comparisons of the host-specific insect-pathogenic fungi within Entomophthoromycotina will likely shed interesting new light on the gene content of these early diverging fungi (De Fine Licht *et al.*, 2016). The presence of unusual genome organization, polyploidy and large genomes in many host-specific insect-pathogenic species within Entomophthoromycotina has previously been a hindrance to genome sequencing (Gryganskyi and Muszewska 2014). However, this study highlights examples of many new proteins and enzymes that may be discovered through genome sequencing and data mining within Entomophthoromycotina.
